# Bidirectional associations between social isolation, loneliness, and cognitive function among Chinese older adults

**DOI:** 10.7189/jogh.15.04077

**Published:** 2025-03-21

**Authors:** Chaoping Pan, Na Cao

**Affiliations:** 1 School of Medical Humanities and Management, Wenzhou Medical University, Wenzhou, China; 2 Key Research Centre of Philosophy and Social Sciences of Zhejiang Province, Institute of Medical Humanities, Wenzhou Medical University, Wenzhou, China

## Abstract

**Background:**

Social isolation (SI), loneliness, and cognitive function (CF) are increasingly acknowledged as significant public health concerns globally. In this study, we aimed to investigate the bidirectional relationships and mediating effects between SI, loneliness, and CF among older adults in China.

**Methods:**

We analysed data from six waves of the Chinese Longitudinal Healthy Longevity Survey conducted between 2002–18. The sample included individuals aged ≥65 years. We used the general cross-lagged panel model to account for confounding factors and reveal mediating effects.

**Results:**

The findings indicated that SI and loneliness can independently lower CF. Moreover, loneliness may lower CF through SI, and SI may also lower CF through loneliness. Finally, we revealed that decreased CF can increase SI and loneliness.

**Conclusions:**

SI and loneliness are significantly intertwined with CF among older adults in China. Interventions aiming at reducing SI, loneliness, and CF should consider the interplay of these factors to enhance the health and well-being of older adults.

Cognitive function (CF) pertains to an individual’s capacity to process information, encompassing attention, memory, executive functioning, and verbal fluency [1,2]. With increasing life expectancy and a global rise in ageing populations, the occurrence of cognitive dysfunction in older adults is on the upswing. Recent research indicates that mild cognitive impairment affects 11.6–19.1% of older adults worldwide [3] and has a prevalence of 15.5% specifically in China [4]. Cognitive dysfunction significantly influences the health of older adults, contributing to conditions such as depression, dementia, disability, diminished quality of life, and even mortality [5–7]. It also places significant financial and caregiving burdens on families and society [8]. Impoverished social relationships are recognised by the Lancet Commission on Dementia Prevention as a significant modifiable risk factor for poor CF in later life, highlighting the need to investigate how they influence CF for effectively enhancing CF among older adults [9].

According to social convoy model, social relationships can be classified into objective and subjective dimensions [10]. Social isolation (SI) and loneliness reflect the objective and subjective facets of impoverished social relationships, respectively [11]. SI denotes the actual absence of social interaction with others and encompasses elements such as living alone, distancing from social ties, and limited social participation [12]. As the global population ages swiftly, SI has emerged as a major concern among older adults. Research reveals that between 10% and 43% of older adults encounter SI in later stages of life [13], with China reporting a particularly high prevalence of 42.4% among older adults [14].

Conversely, loneliness encapsulates a subjective emotion that emerges when there exists a discrepancy between an individual’s desired and actual levels of social connectedness and relationship quality [15]. In countries such as the USA and Europe, approximately 5–40% of older adults experience feelings of loneliness [16], while China records a loneliness rate of 31.3% among older adults [14]. Both SI and loneliness have been demonstrated to independently and detrimentally impact health and well-being, such as frailty, cognitive impairment, disability, and quality of life [17–21].

The bidirectional relationships of SI, loneliness, and depression can be explained by the International Classification of Functioning, Disability and Health (ICF) model [22]. This bidirectional impact may result from distinct underlying mechanisms. For instance, SI might primarily affect CF by reducing intellectual stimulation, while loneliness could predominantly influence CF through psychological distress [23]. The effects of cognitive impairment on SI can be attributed to its constraint on older adults' ability to engage in social interactions [24]. Moreover, the link between loneliness and cognitive impairment may stem from the possibility that cognitive decline could diminish older adults’ capacity to meet their socio-emotional needs [25]. Based on this theory, several studies have indicated that elevated levels of SI and loneliness can independently increase the risk of cognitive impairment among older adults [23,26–28]. Though limited studies explored the bidirectional effect between CF and SI or CF and loneliness among older adults [15,24], previous research has not thoroughly investigated the interplay among SI, loneliness, and CF while adequately accounting for reverse effects and confounding factors. Hence, how these factors interact with each other remains unknown.

The general cross-lagged panel model (GCLM) stands as a robust statistical tool that can well control for the reverse effects and confounding factors when exploring bidirectional relationships and mediating roles in longitudinal data. In this study, we have utilised this approach to explore the bidirectional relationships between SI, loneliness, and CF, and to establish their mediating roles in these relationships. The first hypothesis was that reciprocal relationships exist between SI and CF and between loneliness and CF. Second hypothesis was that SI serves as a mediator for the influence of loneliness on CF, and loneliness acts as a mediator for the impact of SI on CF. Moreover, SI plays a mediating role in the impact of CF on loneliness, while loneliness mediates the impact of CF on SI.

## METHODS

### Data and participants

We drew data from the Chinese Longitudinal Healthy Longevity Survey (CLHLS), a nationally representative longitudinal survey spanning 23 of the 31 provinces in China and encompassing approximately 85% of the total population. Participants included individuals aged ≥65 years. The baseline survey was conducted in 1998, with subsequent data collected in seven waves in 2002, 2005, 2008, 2011, 2014, and 2018. For this research, we used data from six waves of the CLHLS between 2002–18. Additional details about the CLHLS can be found elsewhere [29,30]. Exclusion criteria for this study included individuals aged <65 years at any time point, constituting less than 1% of the total sample. Additionally, as this is a longitudinal study, individuals tracked fewer than twice were excluded. Therefore, the number of respondents for each wave was as follows: n = 8136 (2002), n = 11 427 (2005), n = 11 571 (2008), n = 9194 (2011), n = 6551 (2014), and n = 3469 (2018).

In this study, we employed the multiple imputation (MI) method to address the missing values. The ‘Markov Chain Monte Carlo’ method was specifically employed to conduct five imputations to ensure robust estimates through MI. Five imputations were deemed adequate for producing reliable results using MI. For more details on MI, please refer to another study [31].

### Measures

#### Cognitive function

In line with other studies [32–34], we evaluated CF using the total score derived from the Chinese version of the Mini-Mental State Examination (MMSE), which assesses orientation, registration, food naming, attention and calculation, recall, figure copying, and language skills. These dimensions involve using standardised tests and assessments to measure an individual’s functional capabilities objectively. The total score ranged from zero to 30, with a higher score indicating poorer CF. In this study, we reversed the coding for CF scoring compared to the common MMSE scoring interpretation. This adjustment did not affect the results and was consistent with the assumption that higher SI and loneliness scores were associated with poorer CF outcomes.

#### Social isolation

We applied the five dimensions advocated in earlier literature [14,18,19,35,36] to assess SI among older adults. These dimensions encompassed: 1) living alone, 2) having a spouse, 3) frequent contact with children, 4) frequent contact with siblings, and 5) participating in social activities. Those who lived alone, lacked a spouse, had infrequent contact with children/siblings, or engaged less in social activities were assigned a code of ‘1.’Conversely, individuals who did not live alone, had a spouse, received regular visits from children/siblings, or actively participated in social activities were given a value of ‘0.’ As indicated in previous research [18,19,36], the overall score ranged from zero to five, with a higher score indicating more severe levels of SI.

#### Loneliness

In accordance with prior studies [14,18,35,37,38], we employed a single-item measure to assess loneliness, using the question ‘Do you feel lonely?’ Responses included ‘never’ (zero points), ‘hardly ever’ (one point), ‘sometimes’ (two points), ‘often’ (three points), and ‘always’ (four points), resulting in a total score range of zero to four points. The employment of a single-dimension loneliness scale is prevalent among older populations, and previous research has indicated its robust correlation with multidimensional scales [14,39].

### Analysis

We utilised GCLM to analyse the data, a method recommended by previous studies [40,41]. GCLM has seen increasing use in social and health research, particularly in examining the connections between SI and health [41]. One of the reasons why GCLM is suitable for this analysis is its capacity to model lagged relationships, allowing for the exploration of bidirectional associations and mediating relationships among SI, loneliness and CF in older adults [40,41]. Additionally, GCLM provides advantages in minimising confounding effects and bolstering causal inferences of the relationships between SI, loneliness and CF among older adults by effectively managing stable and time-varying factors [40–42].

To conduct the model analysis, we utilised MPlus, version 8 (Muthén & Muthén, Los Angeles, California, USA) and adhered to the structural equation modelling framework proposed by Zyphur et al [40]. Formally, the model specification used in this study is expressed as:



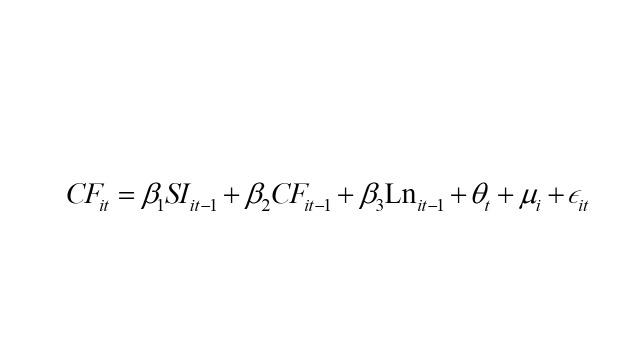





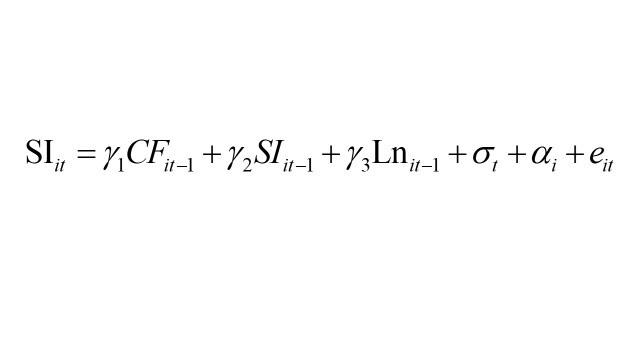





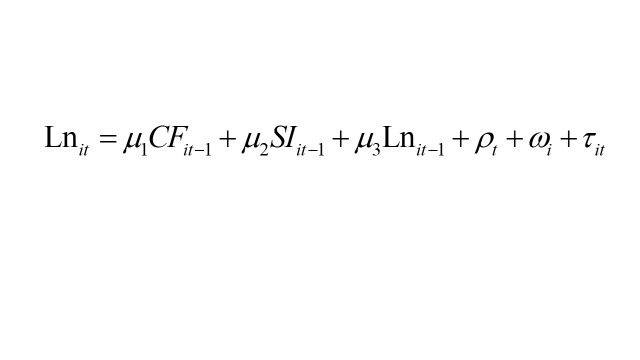



Within the model, the subscripts *i* and *t* denote individuals and time, respectively. SI denotes social isolation, CF represents cognitive function, and Ln indicates loneliness. The regression coefficients to be estimated are β_1_, β_2_, β_3_, γ_1_, γ_2_, γ_3_, μ_1_, μ_2_, and μ_3_. Moreover, θ, σ, and ρ signify time effects, while μ, α, and ώ capture time-invariant effects. ϵ, e, and τ represent individual-specific error terms. It is crucial to recognise that the model does not treat specific time-varying or time-invariant variables as separate entities. Rather, the model manages confounding factors that may vary over time and those that remain constant across time by treating them collectively. This is achieved by including correlation terms between ϵ, e, and τ, as well as μ, α, and ώ to address potential confounding. Previous research has indicated that integrating correlation terms is more effective for controlling confounding factors than including specific confounding variables as covariates [40]. The efficacy of this approach has been increasingly acknowledged and applied in other studies as well [41,43]. It is noteworthy that CF was treated as a continuous variable rather than a dichotomous one, aligning more closely with the gradual nature of CF. The significant results highlight social significance at the population level.

The cross-lagged coefficients β_1_, β_3_, γ_1_, γ_3_, μ_1_, and μ_3_ hold particular significance as they reveal how variations in CF, SI, and Ln at a specific time point predict differences in CF, SI, and Ln at the subsequent time point. The autoregressive paths β_2_, γ_2_, and μ_2_ reflect the extent to which individual differences in expected scores are anticipated by variances from past time points. Additionally, the model enables the computation of mediating effects between SI, loneliness, and CF by evaluating the cross-lagged coefficients. Before conducting the analysis, we standardised the variables, resulting in the regression coefficients being presented as standard deviation (SD) from the mean. This standardisation process aids in comparing different variables used in the analysis. We employed 10 000 bootstrapping methods to compute confidence intervals (CIs). Multiple model fit indices, including the Tucker Lewis index (TLI), the confirmatory fit index (CFI), the root mean square error of approximation (RMSEA), and the standardised root mean squared residual (SRMR) were used to confirm the goodness of model fit of our analysis [40]. TLI and CFI values >0.95 indicated a good model fit. Similarly, RMSEA and SRMR values ≤0.06 were considered to indicate a good fit, while values <0.08 were deemed acceptable [44].

## RESULTS

At baseline, the participants had an average age of 81.83 years. Females made up 54.8% of the sample. Roughly 42.5% of the participants had completed at least one year of education, while a higher proportion (56.1%) resided in rural areas. The participants indicated an average SI score of 2.87 and an average loneliness score of 0.98. The CF was appraised to have an average score of 5.33 (Table S1 in the **Online Supplementary Document**).

The goodness-of-fit statistics for GCLM indicated an excellent fit to the data, with CFI = 0.986, TLI = 0.979, RMSEA = 0.018, and SRMR = 0.031. All values exceed the thresholds for a good fit, suggesting that the model reliably captures the relationships among the variables under investigation (**Table 1**).

**Table 1:** The goodness-of-fit statistics of GCLM (CLHLS, waves 2002–18)

Index	Values
CFI	0.986
TLI	0.979
RMSEA	0.018
SRMR	0.031

CFI – confirmatory fit index, RMSEA – root mean square error of approximation, SRMR – standardised root mean squared residual, TLI – Tucker Lewis index

The analysis revealed that an increase of one SD in SI leads to a future CF enhancement (SD = 0.056; 95% CI = 0.030, 0.316). Conversely, each increase of one SD in loneliness escalated future CF by SD = 0.019 (95% CI = 0.003, 0.143). Moreover, with every one SD increase in CF, SI and loneliness intensified by SD = 0.024 (95% CI = 0.007, 0.119) and SD = 0.049 (95% CI = 0.026, 0.160).

The analysis also demonstrates that elevated levels of SI at a specific time point were associated with amplified loneliness in the subsequent time point (μ_2_ = 0.030; 95% CI = 0.054, 0.203). Conversely, heightened levels of loneliness at a given time point were correlated with increased SI in the subsequent time point (γ_3_ = 0.021; 95% CI = 0.010, 0.089) (**Table 2**).

**Table 2:** The key model parameters of GCLM (CLHLS, waves 2002–18)

Path	Standardised coefficient (95% CI)
SI_t–1_→SI_t_	0.277 (0.250, 0.377)
SI_t–1_→CF_t_	0.056 (0.030, 0.316)
SI_t–1_→Ln_t_	0.030 (0.054, 0.203)
Ln_t–1_→Ln_t_	0.063 (0.041, 0.165)
Ln_t–1_→SI_t_	0.021 (0.010, 0.089)
Ln_t–1_→CF_t_	0.019 (0.003, 0.143)
CF_t–1_→CF_t_	0.154 (–0.556, 0.297)
CF_t–1_→SI_t_	0.024 (0.007, 0.119)
CF_t–1_→Ln_t_	0.049 (0.026, 0.160)

CF – cognitive function, CI – confidence interval, Ln – loneliness, SI – social isolation

The principal mediating effects and their 95% CIs derived from GCLM analysis revealed that the path Ln_t-2_→SI_t-1_→CF_t_ had a standardised coefficient of 0.001 (95% CI = 0.0003, 0.028), indicating that SI might act as a mediating factor in the correlation between CF and loneliness over time. Specifically, increased levels of loneliness at one point in time were linked with elevated levels of SI at the subsequent time point, which consequently was associated with heightened levels of CF in the future.

Furthermore, the pathway SI_t-2_→Ln_t-1_→CF_t_ demonstrated a standardised coefficient of 0.001 (95% CI = 0.0004, 0.024), indicating that SI can also significantly influence CF through loneliness. Specifically, heightened levels of SI at one point in time were associated with increased levels of loneliness at the subsequent time point, which consequently was linked with elevated levels of CF in the future.

Moreover, the pathway CF_t-2_→SI_t-1_→Ln_t_ displayed a standardised coefficient of 0.001 (95% CI = 0.0003, 0.014), suggesting that higher levels of CF at one point in time were correlated with increased levels of loneliness in the future through SI in the subsequent time point. Additionally, the pathway CF_t-2_→Ln_t-1_→SI_t_ exhibited a standardised coefficient of 0.001 (95% CI = 0.0003, 0.014), indicating that higher levels of CF at one point in time were associated with heightened levels of SI in the future through loneliness at the subsequent time point (**Table 3**).

**Table 3:** Key mediating effects from GCLM (CLHLS, waves 2002–18)

Path	Standardised coefficient (95% CI)
Ln_t-2_→SI_t-1_→CF_t_	0.001 (0.0003, 0.028)
SI_t-2_→Ln_t-1_→CF_t_	0.001 (0.0002, 0.029)
CF_t-2_→SI_t-1_→Ln_t_	0.001 (0.0004, 0.024)
CF_t-2_→Ln_t-1_→SI_t_	0.001 (0.0003, 0.014)
SI_t-2_→CF_t-1_→Ln_t_	0.003 (0.001, 0.051)
Ln_t-2_→CF_t-1_→SI_t_	0.0005 (0.00002, 0.017)

CF – cognitive function, CI – confidence interval, Ln – loneliness, SI – social isolation

## DISCUSSION

Based on the social convoy model, we aimed to explore the bidirectional relations and mediating effects among SI, loneliness, and CF among older adults in China using GCLM. Our findings uncovered a reciprocal correlation between SI and CF, as well as a mutual relationship between loneliness and CF, providing support for our initial hypothesis. Additionally, our results demonstrated that SI serves as a mediator for the influence of loneliness on CF, while also playing a mediating role in the impact of CF on loneliness. Conversely, loneliness acts as a mediator for the impact of SI on CF and the impact of CF on SI, validating our secondary hypothesis. These findings align with Lancet Commission on Dementia Prevention which indicated that impoverished social relationships are significant modifiable risk factors for poor CF in later life [9].

The social convoy model indicated that both SI and loneliness can independently interact with CF [10]. Consistent with this theory, the findings of this paper demonstrated that SI and loneliness can independently result in poorer CF. One hypothesis explaining these differing effects is that loneliness and SI may influence CF through distinct pathways [14,38]. For example, SI may primarily impact CF by reducing intellectual stimulation, while loneliness may predominantly affect CF through psychological distress [23]. This outcome aligns with prior literature indicating that both SI and loneliness could affect CF [23,26,28], but contrasts with other studies suggesting that SI, rather than loneliness, could impact CF [32,45]. Our study contributes to the literature by effectively addressing reverse causations within the relationships among SI, loneliness, and CF, as well as appropriately managing confounding factors.

The ICF model also indicated that SI may directly lead to loneliness and subsequently impact CF. Additionally, loneliness can also influence SI by increasing self-centeredness and hypervigilance for social threats, ultimately affecting CF [46]. Consistent with this theory, the present study observed that loneliness may impact CF through SI, while SI may impact CF through loneliness. Previous studies have identified loneliness as a mediator in the relationship between SI and CF [21,47], but have not contemporarily explored the mediating roles of SI in the effects of loneliness on CF. This study contributes to the existing literature by delving into the intricate relationships among SI, loneliness, and CF, thereby extending our comprehension of the mediating effects between these factors. Moreover, the findings indicate that CF can increase SI and loneliness in older adults, likely due to cognitive impairment constraining older adults' social interactions [24], consistent with findings from previous studies [15,24]. This discovery further emphasises the significance of improving CF in interventions designed to mitigate SI and loneliness among older adults.

This study presents several implications. First, the findings demonstrate that both SI and loneliness can independently impact CF. This suggests that interventions like social engagement activities targeting both factors may be beneficial in improving CF. Furthermore, the study suggests that SI could influence CF through its association with loneliness, indicating that interventions like promoting friendships focused on alleviating loneliness might effectively bolster CF in older adults facing SI. Moreover, the research reveals that loneliness can also impact CF through its relationship with SI, implying that interventions like health care services aimed at decreasing SI could efficiently improve CF among older adults experiencing loneliness [10,48]. Lastly, the study emphasises that CF can influence both SI and loneliness, highlighting the importance of preventing SI and loneliness using the interventions, such as cognitive training, among older adults with cognitive impairment. It is crucial to note that in China, family systems and collectivism carry more significance than friendships and social participation compared to western countries [14]. Therefore, these implications may be pertinent to China and other similar cultural contexts that prioritise family systems and collectivism.

This study had several strengths. First, we utilised data from a large, nationally representative sample of individuals aged ≥65 years over a span of 16 years, enabling a more comprehensive exploration of the relationships between CF, loneliness, and SI. Second, we employed a novel statistical method called GCLM, which effectively addressed confounding by reverse causality and controlled for both observable and unobservable time-invariant and time-varying confounds.

We acknowledge certain limitations that need to be addressed. First, the measurement of loneliness relied on a single item, which may not capture the multidimensional nature of this construct comprehensively. Although previous research has shown a strong correlation between single-item and multidimensional loneliness scales [14,39], using a composite measure could have provided a more comprehensive understanding of the relationship between loneliness, SI and CF. Second, constrained by data availability, the study did not include information on participants’ contacts with non-family members, such as friends or neighbours, when measuring SI. To address these limitations, future research endeavours could adopt more inclusive measures of loneliness (*e.g.* University of California Los Angeles loneliness scale) that account for its various dimensions. Moreover, researchers could incorporate data on participants' interactions with friends and non-family members to enhance the precision of SI assessments.

## CONCLUSIONS

In conclusion, we investigated the bidirectional relationships and mediating effects between SI, loneliness, and CF among older adults in China using the innovative GCLM statistical method. We uncovered that SI and loneliness possess independent impacts on CF. Furthermore, loneliness was identified as potentially influencing CF through SI, while SI could also impact CF through loneliness. Lastly, the study highlighted that CF can have an impact on both SI and loneliness. These findings underline the importance of considering the interplay between SI, loneliness, and CF in interventions aimed at enhancing the health and well-being of older adults.

## Additional material


Online Supplementary Document

## References

[1] Zhu M Ding X Wang Q Xue J Shi J Li Z Association between self-perception of aging and cognitive function in Chinese older adults: The mediation effect of health behaviors. Geriatr Nurs. 2023;54:350–6. 10.1016/j.gerinurse.2023.10.016 37967507

[2] Peng TC Chen WL Wu LW Chang YW Kao TW Sarcopenia and cognitive impairment: A systematic review and meta-analysis. Clin Nutr. 2020;39:2695–701. 10.1016/j.clnu.2019.12.014 31917049

[3] Petersen RC Lopez O Armstrong MJ Getchius TSD Ganguli M Gloss D Practice guideline update summary: Mild cognitive impairment: Report of the Guideline Development, Dissemination, and Implementation Subcommittee of the American Academy of Neurology. Neurology. 2018;90:126–35. 10.1212/WNL.0000000000004826 29282327 PMC5772157

[4] Jia LF Du YF Chu L Zhang ZJ Li FY Lyu DY Prevalence, risk factors, and management of dementia and mild cognitive impairment in adults aged 60 years or older in China: a cross-sectional study. Lancet Public Health. 2020;5:e661–e671. 10.1016/S2468-2667(20)30185-7 33271079

[5] Denver P McClean PL Distinguishing normal brain aging from the development of Alzheimer’s disease: inflammation, insulin signaling and cognition. Neural Regen Res. 2018;13:1719–30. 10.4103/1673-5374.238608 30136683 PMC6128051

[6] Aranda MP Kremer IN Hinton L Zissimopoulos J Whitmer RA Hummel CH Impact of dementia: Health disparities, population trends, care interventions, and economic costs. J Am Geriatr Soc. 2021;69:1774–83. 10.1111/jgs.17345 34245588 PMC8608182

[7] Adjoian Mezzaca T Dodds LV Rundek T Zeki Al Hazzouri A Caunca MR Gomes-Osman J Associations Between Cognitive Functioning and Mortality in a Population-Based Sample of Older United States Adults: Differences by Sex and Education. J Aging Health. 2022;34:905–15. 10.1177/08982643221076690 35440227

[8] Jia J Wei C Chen S Li F Tang Y Qin W The cost of Alzheimer’s disease in China and re-estimation of costs worldwide. Alzheimers Dement. 2018;14:483–91. 10.1016/j.jalz.2017.12.006 29433981

[9] Livingston G Huntley J Sommerlad A Ames D Ballard C Banerjee S Dementia prevention, intervention, and care: 2020 report of the Lancet Commission. Lancet. 2020;396:413–46. 10.1016/S0140-6736(20)30367-6 32738937 PMC7392084

[10] Antonucci TC Ajrouch KJ Birditt KS The Convoy Model: Explaining Social Relations From a Multidisciplinary Perspective. Gerontologist. 2014;54:82–92. 10.1093/geront/gnt118 24142914 PMC3894851

[11] Valtorta NK Kanaan M Gilbody S Hanratty B Loneliness, social isolation and social relationships: what are we measuring? A novel framework for classifying and comparing tools. BMJ Open. 2016;6:e010799. 10.1136/bmjopen-2015-010799 27091822 PMC4838704

[12] Holt-Lunstad J Smith TB Baker M Harris T Stephenson D Loneliness and Social Isolation as Risk Factors for Mortality: A Meta-Analytic Review. Perspect Psychol Sci. 2015;10:227–37. 10.1177/1745691614568352 25910392

[13] Nicholson NR A Review of Social Isolation: An Important but Underassessed Condition in Older Adults. J Prim Prev. 2012;33:137–52. 10.1007/s10935-012-0271-2 22766606

[14] Yu B Steptoe A Chen YJ Social isolation, loneliness, and all-cause mortality: A cohort study of 35,254 Chinese older adults. J Am Geriatr Soc. 2022;70:1717–25. 10.1111/jgs.17708 35229887

[15] Cachón-Alonso L Hakulinen C Jokela M Komulainen K Elovainio M Loneliness and Cognitive Function in Older Adults: Longitudinal Analysis in 15 Countries. Psychol Aging. 2023;38:778–89. 10.1037/pag0000777 37856398

[16] Leigh-Hunt N Bagguley D Bash K Turner V Turnbull S Valtorta N An overview of systematic reviews on the public health consequences of social isolation and loneliness. Public Health. 2017;152:157–71. 10.1016/j.puhe.2017.07.035 28915435

[17] Freak-Poli R Ryan J Tran T Owen A Power JM Berk M Social isolation, social support and loneliness as independent concepts, and their relationship with health-related quality of life among older women. Aging Ment Health. 2022;26:1335–44. 10.1080/13607863.2021.1940097 34219569

[18] Guo L An L Luo F Yu B Social isolation, loneliness and functional disability in Chinese older women and men: a longitudinal study. Age Ageing. 2021;50:1222–8. 10.1093/ageing/afaa271 33352582

[19] Jarach CM Tettamanti M Nobili A D’Avanzo B Social isolation and loneliness as related to progression and reversion of frailty in the Survey of Health Aging Retirement in Europe (SHARE). Age Ageing. 2021;50:258–62. 10.1093/ageing/afaa168 32915990 PMC7793602

[20] Berkman LF Glass T Brissette I Seeman TE From social integration to health: Durkheim in the new millennium. Soc Sci Med. 2000;51:843–57. 10.1016/S0277-9536(00)00065-4 10972429

[21] Yang R Wang H Edelman LS Tracy EL Demiris G Sward KA Loneliness as a mediator of the impact of social isolation on cognitive functioning of Chinese older adults. Age Ageing. 2020;49:599–604. 10.1093/ageing/afaa020 32147683

[22] World Health Organization. international classification of functioning, disability and health. 2001. Available: https://www.who.int/classifications/international-classification-of-functioning-disability-and-health. Accessed: 18 September 2024.

[23] Tang F Li K Wang Y Zhu Y Jiang Y Social Disconnectedness, Perceived Loneliness, and Cognitive Functioning: The Role of Neighborhood Environment. Innov Aging. 2024;8:igae009. 10.1093/geroni/igae009 38500713 PMC10946307

[24] Qi X Pei YL Malone SK Wu B Social Isolation, Sleep Disturbance, and Cognitive Functioning (HRS): A Longitudinal Mediation Study. J Gerontol A Biol Sci Med Sci. 2023;78:1826–33. 10.1093/gerona/glad004 36617184 PMC10562894

[25] Zhong BL Chen SL Tu X Conwell Y Loneliness and Cognitive Function in Older Adults: Findings From the Chinese Longitudinal Healthy Longevity Survey. J Gerontol B Psychol Sci Soc Sci. 2017;72:120–8. 10.1093/geronb/gbw037 27013536 PMC5156491

[26] Lara E Caballero FF Rico-Uribe LA Olaya B Haro JM Ayuso-Mateos JL Are loneliness and social isolation associated with cognitive decline? Int J Geriatr Psychiatry. 2019;34:1613–22. 10.1002/gps.5174 31304639

[27] Fang F Hughes TF Weinstein A Dodge HH Jacobsen EP Chang CCH Social Isolation and Loneliness in a Population Study of Cognitive Impairment: The MYHAT Study. J Appl Gerontol. 2023;42:2313–24. 10.1177/07334648231192053 37518906 PMC10825064

[28] Souza JG Farias-Itao DS Aliberti MJR Bertola L Andrade FBD Lima-Costa MF Social Isolation, Loneliness, and Cognitive Performance in Older Adults: Evidence From the ELSI-Brazil Study. Am J Geriatr Psychiatry. 2023;31:610–20. 10.1016/j.jagp.2023.03.013 37211500

[29] Zimmer Z Martin LG Nagin DS Jones BL Modeling Disability Trajectories and Mortality of the Oldest-Old in China. Demography. 2012;49:291–314. 10.1007/s13524-011-0075-7 22246796

[30] Gao M Sa Z Li Y Zhang W Tian D Zhang S Does social participation reduce the risk of functional disability among older adults in China? A survival analysis using the 2005-2011 waves of the CLHLS data. BMC Geriatr. 2018;18:224. 10.1186/s12877-018-0903-3 30241507 PMC6151053

[31] Schafer JL. Analysis of Incomplete Multivariate Data. New York, New York, USA: Chapman & Hall; 1997.

[32] Yu B Steptoe A Chen Y Jia X Social isolation, rather than loneliness, is associated with cognitive decline in older adults: the China Health and Retirement Longitudinal Study. Psychol Med. 2021;51:2414–2421. 10.1017/S0033291720001014 32338228

[33] Yao Y Lv XZ Qiu CX Li JJ Wu X Zhang H The effect of China’s Clean Air Act on cognitive function in older adults: a population-based, quasi-experimental study. Lancet Healthy Longev. 2022;3:e98–e108. 10.1016/S2666-7568(22)00004-6 35224526 PMC8881012

[34] Qin A Chen C Bao B Xin T Xu L Estimating the impact of different types hearing loss on cognitive decline and the joint effect of hearing loss and depression on cognitive decline among older adults in China. J Affect Disord. 2024;351:58–65. 10.1016/j.jad.2024.01.203 38286235

[35] Huang Y Zhu X Liu X Li J The effects of loneliness, social isolation, and associated gender differences on the risk of developing cognitive impairment for Chinese oldest old. Aging Ment Health. 2023;27:1360–67. 10.1080/13607863.2022.2116396 36065623

[36] Steptoe A Shankar A Demakakos P Wardle J Social isolation, loneliness, and all-cause mortality in older men and women. Proc Natl Acad Sci U S A. 2013;110:5797–801. 10.1073/pnas.1219686110 23530191 PMC3625264

[37] Courtin E Knapp M Social isolation, loneliness and health in old age: a scoping review. Health Soc Care Community. 2017;25:799–812. 10.1111/hsc.12311 26712585

[38] Tanskanen J Anttila T A Prospective Study of Social Isolation, Loneliness, and Mortality in Finland. Am J Public Health. 2016;106:2042–8. 10.2105/AJPH.2016.303431 27631736 PMC5055788

[39] Luo Y Waite LJ Loneliness and Mortality Among Older Adults in China. J Gerontol B Psychol Sci Soc Sci. 2014;69:633–45. 10.1093/geronb/gbu007 24550354 PMC4049147

[40] Zyphur MJ Allison PD Tay L Voelkle MC Preacher KJ Zhang Z From Data to Causes I: Building A General Cross-Lagged Panel Model (GCLM). Organ Res Methods. 2020;23:651–87. 10.1177/1094428119847278

[41] Del Pozo Cruz B Perales F Alfonso-Rosa RM Del Pozo-Cruz J Bidirectional and Dynamic Relationships Between Social Isolation and Physical Functioning Among Older Adults: A Cross-Lagged Panel Model of US National Survey Data. J Gerontol A Biol Sci Med Sci. 2021;76:1977–80. 10.1093/gerona/glab110 33839792

[42] Hamaker EL Kuiper RM Grasman RPPP A Critique of the Cross-Lagged Panel Model. Psychol Methods. 2015;20:102–16. 10.1037/a0038889 25822208

[43] Pan C Cao N Bidirectional and dynamic relationships between social isolation and frailty among older adults in China. Arch Gerontol Geriatr. 2023;116:105229. 10.1016/j.archger.2023.105229 39491076

[44] Santini ZI Jose PE Cornwell EY Koyanagi A Nielsen L Hinrichsen C Social disconnectedness, perceived isolation, and symptoms of depression and anxiety among older Americans (NSHAP): a longitudinal mediation analysis. Lancet Public Health. 2020;5:e62–e70. 10.1016/S2468-2667(19)30230-0 31910981

[45] Griffin SC Mezuk B Williams AB Perrin PB Rybarczyk BD Isolation, Not Loneliness or Cynical Hostility, Predicts Cognitive Decline in Older Americans. J Aging Health. 2020;32:52–60. 10.1177/0898264318800587 30289338

[46] Cacioppo JT Cacioppo S Loneliness in the Modern Age: An Evolutionary Theory of Loneliness (ETL) - ScienceDirect. Adv Exp Soc Psychol. 2018;58:127–97. 10.1016/bs.aesp.2018.03.003

[47] Khalaila R Cohn-Schwartz E Shiovitz-Ezra S Lawlor B A prospective association between social isolation and cognitive performance among older adults in Europe: the role of loneliness and poor oral health. Aging Ment Health. 2024;28:1162–8. 10.1080/13607863.2023.2299968 38192062

[48] Martin PR Reece J Lauder S McClelland A A Randomised Controlled Trial of a Social Support Intervention. Appl Psychol Health Well-Being. 2011;3:44–65. 10.1111/j.1758-0854.2010.01044.x

