# Supplementary material for: Bidirectional associations between social isolation, loneliness, and cognitive function among Chinese older adults
Source: J Glob Health. 2025 Mar 21;15:04077. doi: 10.7189/jogh.15.04077 (PMC11931458; doi:10.7189/jogh.15.04077)
Supplement: Online Supplementary Document [file jogh-15-04077-s001.pdf]

Supplement to: Pan C, Cao N. Bidirectional associations between social isolation, loneliness, and cognitive function among Chinese older adults. J Glob Health. 2025;15:04077.

Table S1 baseline characteristics of sample in 2002

Variable	Mean (SD) or %
SI	2.87 (1.01)
Loneliness	0.98(0.99)
CF	5.33 (6.89)
Age	81.83 (10.87)
Sex, female (%)	54.8
Education, received at least one year of education (%)	42.5
Residence, rural (%)	56.1

Note: SD = Standard Deviation; SI=Social Isolation; CF=Cognitive Function.
